# Rural Resident Experience on National Basic Public Health Services: A Cross-Sectional Survey in 10 Western Provinces of China

**DOI:** 10.3390/healthcare7040160

**Published:** 2019-12-07

**Authors:** Jinlin Liu, Ying Mao

**Affiliations:** 1 Research Center for the Belt and Road Health Policy and Health Technology Assessment, Xi’an Jiaotong University, Xi’an 710049, China; 2 School of Public Policy and Administration, Xi’an Jiaotong University, Xi’an 710049, China

**Keywords:** resident experience, satisfaction, basic public health services, rural areas, China

## Abstract

National basic public health services (BPHSs) are important for promoting the health of rural populations. A better understanding of rural BPHSs from the viewpoint of residents utilizing the services can help health-related departments and primary health care (PHC) centers further improve rural BPHSs. By conducting a large-scale cross-sectional survey in 10 western provinces of China, the study depicts rural resident experiences with rural BPHSs. Of the 9019 participants, 59.33% and 66.48% did not receive services related to health examinations or health education in the six months prior to the survey, respectively. A total of 56.90% were satisfied with the rural BPHSs, and the mean overall satisfaction score was 3.61 ± 0.908 (out of a maximum of 5). The most satisfying domain for rural residents with BPHSs was the attitude of PHC workers, whereas rural residents with chronic diseases were the least satisfied with the health management. Satisfaction with the attitude of PHC workers was identified as the strongest determinant of rural residents’ overall satisfaction with BPHSs. This study could enlighten rural BPHSs management in China.

## 1. Introduction

In 1978, the Declaration of Alma-Ata was firstly put forward by leaders from around the world to call for urgent and effective national and international action to develop and implement primary health care (PHC) throughout the world. After 40 years, in 2018, the Declaration of Astana reaffirmed PHC as the most effective and efficient approach to achieve universal health coverage and the health-related Sustainable Development Goals [1]. Focusing on PHC, China has made extensive effort in this regard, and in 2009, launched the new health-care reform plan [2]. Two of the five major targets in the action plan related to PHC were the improvement of medical care and public health service system at a grassroots level and the promotion of the basic public health services (BPHSs). As the new health-care reform continues and new challenges emerge, China put forward and endorsed a national strategy in 2015 known as “Healthy China,” to promote the realization of the goals of the new health-care reform and improve population health [3]. In the strategy it further emphasized the principle of prevention first and then a combination of prevention and treatment (“Yu-Fang-Wei-Zhu, and Fang-Zhi-Jie-He” in Chinese). Thus, the public health system, especially the BPHSs in China, plays an increasingly important role in disease prevention and population health protection and improvement in national policy contexts.

China’s new health-care reform in 2009 was anchored in five interdependent areas, among which, the goal related to BPHSs was to make them available and equal for all [4]. A BPHSs package was provided for the residents free of charge [5]. The initial package in 2009 included nine categories, i.e., establishment of health archives for all residents, health education, immunization for children, health management for children, maternal health management, elderly health management, health management for residents with chronic diseases, reporting and handling of infectious diseases, and health management for residents with severe mental illness [2,6]. According to the policy from the National Health Commission of China in 2019 [7], the latest BPHS package includes 12 categories that covers health management for residents with tuberculosis, traditional Chinese medicine health management, and health supervision assistance are added into the initial 2009 package. In addition, some other major public health services and family planning program are included in the latest package. The BPHSs providers are primarily from health-care centers, including community health centers (CHCs) and stations in urban areas and township health centers (THCs) and village clinics in rural areas [8]. In 2019, the government provided 69 Yuan ($9.85) per head (raised from 15 Yuan, i.e., $2.20, in 2009) for PHC providers to deliver BPHSs. The central government directed its share of subsidy towards western and central areas [4], which means the rich eastern provinces bear most of the subsidy funding. The delivery of BPHSs was highly influenced by public subsidies from the government [9]. Meanwhile, to further encourage PHC providers to improve BPHSs, the budget decided by capitation is tied to an annual performance assessment [4]. Additionally, in rural areas, the village doctors are administered by the THCs, and the THCs are in the charge of public health funding and are required to allocate no less than 40% of the money to village clinics [10].

Previous studies have been conducted focusing on BPHSs in China. A review study of 10 years of health-care reform of progress by Yuan et al. reported that the policies of BPHSs had increased the BPHSs’ coverage and reduced disparities between areas of higher and lower economic development in China, although the progress varies among different services in the package [6]. Zhang et al. found that the BPHSs’ coverage for internal migrants was greatly improved and benefited from increasing government subsidies [11]. By reviewing the development and reform of public health in China from 1949 to 2019, Wang et al. pointed out that BPHSs currently faced several challenges, which included that the quality of BPHS quality was yet to be improved, there was poor integration among different services, a lowered efficiency in the delivery system, a shortage of well-trained public health workers, and other sectors in the government were not fully engaged [12]. Both Wang et al. [13] and Yin et al. [14] found that the actual cost per head for BPHSs’ provision was higher than the average compensation per capita in China. The challenges in delivering BPHSs by village doctors were identified, which included heavy workload, poor working conditions, a shortage and gender imbalance of village doctors, low income or subsidies, lack of social security, inadequate professional training, and insufficient cooperation with rural residents [10,15,16,17,18]. Liang et al. found that the shortage of qualified public health workers in PHC centers was the bottleneck for BPHSs delivery in Southwest China [19]. Similar challenges were identified in BPHSs’ delivery by health workers in CHCs of urban areas, which included a shortage of primary health workers and public subsidies, as well as insufficient professional skills [20].

Given that the policy of BPHSs in China does not pay enough attention to the BPHSs’ quality [4,6] and the BPHSs’ delivery faces more challenges in rural areas, the purpose of this study is to explore the experiences or attitudes of rural residents toward BPHSs. Similar to the relation between patients’ satisfaction and quality of medical services [21], the evaluation based on resident experience could provide real and effective feedback on the quality of rural BPHSs to some extent. Therefore, based on a large-scale cross-sectional survey among rural residents in 10 western provinces of China, this study aimed to depict their experience of rural BPHSs and serve as a reference for related policymakers to have a more comprehensive understanding on policy implementation. To our knowledge, our study is the first to conduct a large-scale survey among rural residents that covers 10 western provinces in China to analyze their experience with rural BHPSs.

The rest of the paper is divided as follows. The next section outlines the research methodology, then the results of this study and discussion are presented, and finally the paper comes to a conclusion.

## 2. Materials and Methods

### 2.1. Study Design and Participants

A mixed-method research was conducted, which included quantitative and qualitative designs to identify the experience of rural residents with basic public health services. Specifically, for quantitative design, a structured questionnaire was developed, and as a complement to quantitative evidences, a qualitative study was introduced which used in-depth interviews with open-ended questions. Conducting a household survey was difficult due to rural residents’ distrust of investigators unknown to the rural community, therefore we chose to carry out the survey in rural medical institutions. Firstly, as mentioned above, all residents were involved in BPHSs programs, and were patients seeking medical services in rural medical institutions and thus had experience with BPHSs. Secondly, under the permission and support from rural medical institutions, these selected patients would more likely be able to accept the invitation to complete the survey.

The cross-sectional survey based on questionnaires was carried out simultaneously in 10 provinces in Western China, which included Gansu, Guangxi, Kweichow, Inner Mongolia, Qinghai, Shaanxi, Sichuan, Tibet, Xinjiang, and Yunnan, and the interviews were further conducted only in Shaanxi. 10 teams in total from the 10 provinces participated in the field work coordinated by Xi’an Jiaotong University in Shaanxi.

**Figure 1:**
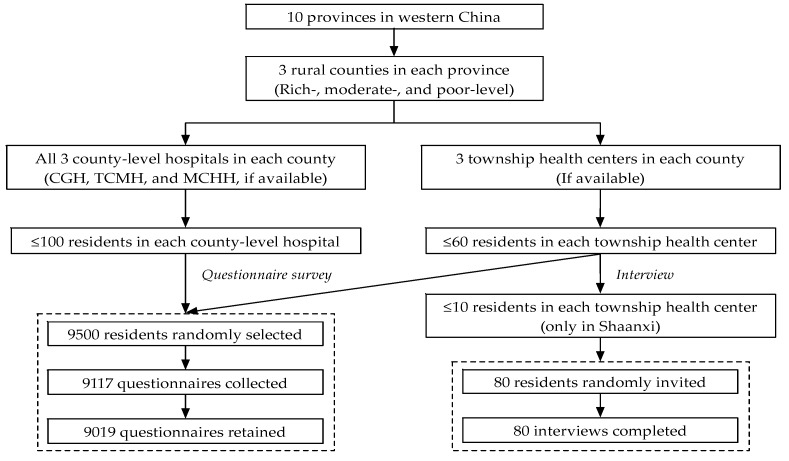
Study profile. CGH: County general hospital; TCMH: Traditional Chinese medical hospital; and MCHH: Maternal and child health hospital.

A three-stage random sampling method was used. Figure 1 shows the complete sampling process. First, in each province, 3 rural counties were randomly selected (i.e., rich-, moderate-, and relatively poor-level) according to the stratification criteria regarding the economic development level of all rural counties. Second, in general, every rural county in China has several township health centers (THCs) and 3 county-level hospitals which include 1 county general hospital (CGH), one traditional Chinese medicine hospital (TCMH), and one maternal and child health hospital (MCHH). We invited all 3 county-level hospitals and randomly selected 3 THCs (if available) to participate, and the survey was conducted after getting approval from these invited medical institutions. Thirdly, in terms of resident selection, for the survey-based questionnaire, no more than 100 residents undergoing medical services in each county-level hospital and no more than 60 residents undergoing medical services in each THC were randomly selected. For the interview survey, as introduced before, we only conducted it in THCs in Shaanxi, and no more than 10 from the 60 residents who had been selected to fill in the questionnaires were randomly invited to attend further interviews. The sample sizes for questionnaire and interview survey were determined by all co-PIs (Principal Investigators) in 10 research teams and two experts from the World Health Organization (WHO) under consideration of the study objective, duration, and budget, etc.

Approximately 9500 residents undergoing medical services in total were randomly selected from rural medical institutions in 10 western provinces of China. Among which, 9117 (with a response rate of 95.97%) accepted the invitation and completed the questionnaires however, after data integration and cleaning, 98 questionnaires were excluded because of missing values regarding overall satisfaction with the basic public health services and 9019 questionnaires were retained and included in the study. In addition, 80 residents undergoing medical services in THCs of Shaanxi were further invited to attend the interviews and all of them completed the interviews.

### 2.2. Data Collection and Variables Measurement

A brief questionnaire and interview outline were designed initially according to the research objective by the research team in Xi’an Jiaotong University. Then each of the other 9 research teams reviewed them by group discussions. During the process, small-scale pre-surveys had been implemented among rural residents to test the logic and rationality of the questionnaire and interview outline. Finally, they were finalized through group discussion by all co-PIs. Two WHO experts also gave their feedback.

The survey was conducted during June to December 2013 simultaneously in 10 western provinces. Before the survey, every investigator received the standardized training that included an understanding of the survey’s purpose and content and mastering some related survey skills. Each questionnaire would take about 10 minutes and all questionnaires were completed by investigators through face-to-face Q and A with patients in rural medical institutions. Additionally, in-depth interviews were conducted, recorded, and transcribed by the investigators. As Figure 1 shows, the interviews were conducted only in a small sample in THCs in Shaanxi based on two considerations, i.e., first, the THCs played the most important role in the BPHSs’ delivery for rural residents, and second, as this study focused on quantitative analyses, small-scale qualitative data filled in the gaps observed in the quantitative data and complemented the quantitative analysis.

The quantitative data extracted from questionnaire survey consisted of two sections. The first was rural residents’ general sociodemographic characteristics which included 6 variables: (1) Gender was coded as ‘female’ and ‘male’; (2) age was originally a continuous variable and was categorized as ‘≤35 years’, ‘36–45 years’, and ‘≥ 46 years’; (3) education attainment was categorized as ‘illiteracy’, ‘primary school’, ‘junior high school’, and ‘senior high school or above’; (4) occupation was coded as ‘farmer’, ‘nonfarmer’ (i.e., worker, teacher, and student etc.), and ‘unemployed or retired’; (5) monthly income was grouped as ’no regular income’, ‘≤2000 Yuan’, and ‘≥2001 Yuan’; (6) health status was measured by the question of ‘Do you have a chronic disease that was diagnosed by a doctor in the past six months prior to the survey?’. Two responses were provided with ‘no’ or ‘yes’. If the resident had a chronic disease, his or her health status was defined as relatively bad, and if there is no chronic disease, the health status was good.

The second part was related to resident experience with BPHSs in rural areas. As introduced before, the national initial BPHSs package included 9 categories, and considering the difficulty of access to patients with severe mental illness, the study included only other 8 categories of the initial BPHSs package. Specifically, we first asked residents that whether they received health examination and health education services in past 6 months prior to the survey, then measured their satisfaction with the BPHSs regarding health education, immunization, health management, chronic diseases management (i.e., hypertension and diabetes), reporting and handling of infectious diseases, PHC worker’s attitude, and the overall satisfaction. For satisfaction with services that focused on key groups of people, if the participants did not have direct experience on them, they were asked to report their feeling based on the experience of their families, friends, or neighbors. The Likert five-point scales were used to measure rural residents’ satisfaction with BPHSs, and each variable was given the statement ‘strongly dissatisfied’, ‘dissatisfied’, ‘neither dissatisfied nor satisfied’, ‘satisfied’, or ‘strongly satisfied’, with a score of 1, 2, 3, 4, or 5 assigned to each scale, respectively.

In addition, qualitative data were collected from 4 open-ended questions to analyze the actual implementation situation and value of BPHSs as a complement, and identify the existing problems and potential reasons of rural BPHSs from residents’ views.

### 2.3. Statistical Methods

Cronbach’s α was used to test the internal consistency and reliability of the questionnaire in terms of rural resident satisfaction with BPHSs. The categorical variables were displayed using ‘number’ and ‘percentage’. Continuous variables were described using ‘mean’ and ‘interquartile range (IQR)’ if they presented an abnormal distribution tested by the One-Sample Kolmogorov–Smirnov method. Meanwhile, the variables related to rural resident satisfaction with BPHSs were also displayed by ‘mean’ score and ‘standard deviation (S.D.)’.

One-way ANOVA was conducted to assess the difference in the mean score of overall satisfaction with rural BPHSs in different groups of sociodemographic characteristics of rural residents. Spearman correlation analyses were adopted to identify the correlation between overall satisfaction of residents with rural BPHSs and the satisfaction score of each indicator. In addition, multiple linear regression (MLR) and binary logistic regression (BLR) were both applied to identify influencing factors associated with rural residents’ overall satisfaction with BPHSs. For MLR analyses, resident’s overall satisfaction score was set as the dependent variable, independent variables included those specific indictors related to resident satisfaction with rural BPHSs, and all sociodemographic characteristics were introduced as the control variables. For BLR, independent variables and control variables were consistent with those set in the MLR model, while dependent variable, i.e., resident’s overall satisfaction with BPHSs, was converted into a binary variable, specifically, ‘strongly satisfied’ and ‘satisfied’ were defined as the ‘positive answers’ (1 = satisfied), and ‘neither dissatisfied nor satisfied’, ‘dissatisfied’, and ‘strongly dissatisfied’ were set as ‘negative answers’ (0 = dissatisfied). One thing to note was that the independent variables and control variables introduced in the multivariate analyses were those that had been significant in prior univariate analyses, i.e., one-way ANOVA or Spearman correlation analyses. Significance level was set at *p*-value < 0.05. All the data analyses were completed in the Statistical Package for Social Sciences 24.0 (SPSS, IBM, Armonk, NY, USA) for MAC.

In addition, the scissor-and-sort technical was adopted to analyze the qualitative data [22]. We used an inductive approach to extract and sum up the topics mentioned in the interviews and data that was most relevant to the main research question. All the qualitative analysis was conducted manually without using any software of qualitative analysis.

### 2.4. Charactersitics of Participants

A total of 9019 residents who completed the questionnaires were included in this study. Of the 9019 participants, 80 in THCs of Shaanxi further participated in interviews. Table 1 shows their characteristics. Among all these participants, 56.55% were females. The median age was 39 years (IQR: 28–52 years) and 63.17% were younger than 42.38 years. A total of 68.40% attained the education of junior high school or below. A total of 47.01% of the participants were farmers. A total of 31.22% had no regular income per month, and only 26.86% received a monthly income of more than 2001 Yuan. In addition, 26.29% of participants had a chronic disease diagnosed by a doctor in the past 6 months prior to the survey.

### 2.5. Ethics

The study was approved by Ethics Committee of School of Medicine in Xi’an Jiaotong University (China), and the approval number was 2014189. All surveys including questionnaires and interviews were anonymous and investigators obtained verbal informed consents from all participants. Data used in this study were available upon request.

## 3. Results

### 3.1. Resident Experience on Rural Basic Public Health Services

The interview results revealed that only 63 of the 80 interviewees (78.75%) fully knew the policies of rural BPHSs, and others just understood a few of the services. In terms of establishment of health archives, all interviewees reported that they had the archives however, some interviewees thought they were of less value, for example:

“My health archive was established several years ago, but I have never used it and when I go to a doctor, they don’t need my health archive.” (SC101)

“I can know my health status from my health archive, but it doesn’t seem to be updated.” (SC205)

**Figure 2:**
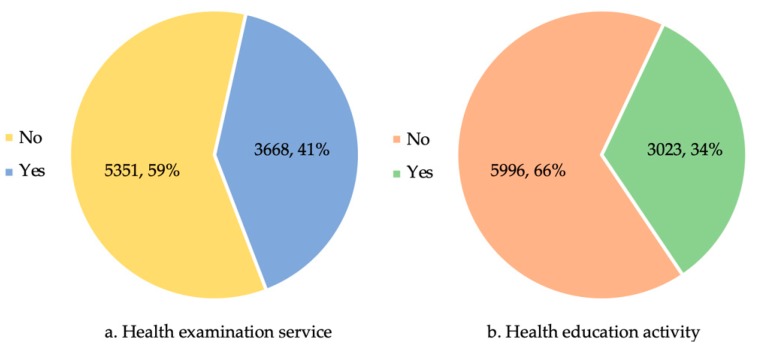
Experience of health examination and health education services in the past six months prior to the survey. (**a**) for health examination service and (**b**) for health education activity.

As Figure 2 shows, only 40.67% and 33.52% of residents had received a health examination service and participated in the health education activities in the past six months prior to the survey, respectively. More than 60% of rural residents did not receive services related to a health examination or health education. In terms of the potential reasons of low utilization of these services, they could be attributed to poor health consciousness, poor geographic accessibility, and shortage of PHC workers based on the interview results.

“I don’t like to take part in these activities as I think they are useless.” (SC103)

“My home is far from the village clinic and THC, and that’s very difficult for PHC workers to provide these services frequently.” (SC301)

“I know the PHC workers need to provide the services for all rural residents, but there are very few PHC workers.” (SC110)

The Cronbach’s α of Likert five-point scales on resident satisfaction with rural BPHSs were 0.912, which shows positive internal consistency and acceptable reliability of the questionnaires as the value was >0.7. Table 2 shows the results of resident satisfaction with BPHSs in rural areas of China. A total of 56.90% of rural residents were satisfied (i.e., satisfied or strongly satisfied) overall with BPHSs, and the mean overall satisfaction score was 3.61 ± 0.908 out of a maximum of 5. Health management for residents with chronic diseases was the domain that rural residents were least satisfied with (3.38 ± 0.999) and immunization and health management for children was the most satisfying domain (3.56 ± 0.922) among the BPHSs package. Besides, the mean satisfaction score towards PHC worker’s attitude was 3.64 ± 0.910, which was higher than all the satisfaction scores of rural BPHSs in the package. All indicators regarding rural residents’ satisfaction with the BPHSs scored >3.00.

Meanwhile, residents’ overall satisfaction with rural BPHSs significantly correlated with all seven satisfaction indictors (i.e., satisfaction with health education, etc.). Specifically, satisfaction with PHC worker’s attitude (r = 0.710, *p* < 0.001) and satisfaction with immunization and health management for children (r = 0.521, *p* < 0.001) had the highest Spearman correlation and least correlation, respectively, with residents’ overall satisfaction with rural BPHSs.

In addition, Table 3 shows that the age (*p* < 0.001), education (*p* < 0.05), occupation (*p* < 0.001), monthly income (*p* < 0.001), and health status (*p* < 0.001) significantly correlated with residents’ overall satisfaction with rural BPHSs based on the one-way ANOVA.

### 3.2. Influencing Factors of Residents’ Overall Satisfaction with Rural Basic Public Health Services

The results of univariate analyses in Table 2 identify the significant positive correlations between residents’ overall satisfaction with rural BPHSs and all seven satisfaction indicators (*p* < 0.001), i.e., the higher the score of each of the seven satisfaction indictors, the higher the residents’ overall satisfaction score. Meanwhile, results in Table 3 show that the residents who reported a significantly higher score of overall satisfaction with rural BPHSs were those who were older than 35 years, those who received an education of primary school, those who had an occupation as a farmer, those who received a regular income per month (i.e., ≤2000 or ≥2001 Yuan), and those who had a relatively bad health status.

In addition, MLRs and BLRs were conducted to further identify influencing factors of residents’ overall satisfaction with rural BPHSs. Table 4 presents all the results. Results of the MLRs were consistent with BLRs’ results and all show that residents’ overall satisfaction with rural BPHSs was significantly associated with their satisfaction with health education, immunization and health management for children, maternal health management, elderly health management, health management for residents with chronic diseases, reporting and handling of infectious diseases, and PHC worker’s attitude, and age of residents. Taking the results of BLRs as the example for explanation, a higher satisfaction with health education (OR: 1.38; 95% CI: 1.26–1.51), immunization and health management for children (OR: 1.19; 95% CI: 1.08–1.30), maternal health management (OR: 1.33; 95% CI: 1.21–1.46), elderly health management (OR: 1.33; 95% CI: 1.22–1.46), health management for residents with chronic diseases (OR: 1.37; 95% CI: 1.25–1.49), reporting and handling of infectious diseases (OR: 1.49; 95% CI: 1.36–1.63), attitude of PHC worker (OR: 4.61; 95% CI: 4.17–5.08) would significantly increase residents’ overall satisfaction with rural BPHSs. Meanwhile, residents who were 46 years or older were significantly more satisfied with overall rural BPHSs (OR: 1.33; 95% CI: 1.14–1.55) than those younger than 35 years.

## 4. Discussion

From a global perspective, there has been a consensus on the importance of public health for the improvement and protection of a population’s health, and in addition it is an essential component in any country’s health care system and policies. Public health related reports or recommendations can be found in the European Union [23], U.K. [24,25], U.S. [26], and WHO [27]. Although public health policies or services differ from country to country because of the different disease spectrum and health status of populations in these countries, they will generally pay attention to key parts of the population like the elderly, women, children, those with chronic diseases and infectious diseases, etc. In China, the national BPHSs are different than those in other countries, thus there is little international evidence that can be used for direct comparison with the findings in our study. Consequentially, we focus on domestic research evidence in China.

The study indicates that rural residents’ utilization of the BPHSs was relatively low. A total of 59.33% and 66.48% of participants did not receive any health examination services and health education services in the past six months prior to the survey, respectively. The interviews further provided explanations, i.e., unwillingness to participate, poor geographic accessibility, and a shortage of PHC workers, which were consistent with prior studies. Both Wang et al. [15] and Zhang et al. [16] have pointed out these challenges in the provision of rural BPHSs. Firstly, the unwillingness of rural residents to participate in rural BPHSs and the insufficient cooperation with PHC workers might be due to poor health consciousness of rural residents and worthless contents and quality of the BPHSs cognized by rural residents. Secondly, the shortage of PHC workers in rural areas has been a perennial problem in China. The rural medical institutions, especially in THCs, have found it difficult to attract medical graduates [28,29] and retain existing health workers [30,31]. Although China has implemented policies such as the rural-oriented tuition-waived medical education program to address these challenges, its effect would not be significant in the short term [32]. Meanwhile, because of the lack of professional public health workers, PHC workers need to provide medical services and BPHSs at the same time, which brings a heavy workload and low job satisfaction [33]. Thirdly, due to the unique geographic situation of western rural areas in China, rural residents are scattered and may live far from THCs and village clinics, which makes it difficult for them to receive BPHSs. Similar results were reported in terms of spatial accessibility to BPHSs in South Sudan [34]. All the above challenges should be addressed in the future implementation of rural BPHSs, thus to further protect the health of rural population.

The results showed that only 56.90% of residents were satisfied with rural BPHSs and the mean overall satisfaction score was 3.61 ± 0.908 out of a maximum of 5, both of which indicated that the satisfaction of rural residents with the BHPSs was not very high. In general, rural residents’ satisfaction with the BPHSs varied considerably among different studies in China. Wei et al. reported that 95.94% of rural residents in Sichuan were satisfied with the BPHSs [35], Liu et al. found 93.80% of rural residents with hypertension were satisfied with the chronic disease management service [36], Xu et al. reported the satisfaction rate of 98.10% among rural residents with the BPHSs [37], and similar results on a higher satisfaction of rural residents with the BPHSs, compared with the results in our study, have been identified by Deng et al. (86.6%) [38], Qiu et al. (86.73%) [39], Liu et al. (91.69%) [40], Zhou et al. (62.93%) [41], Shang et al. (93.84%) [42], Huang et al. (69.40%) [43], and He et al. (76.80%) [44]. Meanwhile, Xu et al. found that only 37.96% of rural residents in Anhui were satisfied with BPHSs [45], and Zhang et al. identified a low satisfaction of 33.91% among rural residents in Shandong [46]. Both these results were lower than the satisfaction rate among rural residents in our study. In addition, urban residents had a higher satisfaction, for example, Ji et al. [47] and Hao et al. [48] identified satisfaction rates of 87.20% and 75.19% among urban residents with BPHSs in Anhui and Guangdong, respectively. Compared with these existing studies, our study focused on western rural residents and had a good sample representation to some extent based on a large sample, so the findings in our study could better reflect rural resident experience with BPHSs in Western China. Internationally, Gebreyesus found that 41.7% of rural clients were satisfied with family planning services in public health facilities in a town of Eastern Ethiopia [49], Gitobu et al. reported a satisfaction rate of 54.5% of mothers with free maternal healthcare services provided by public health facilities in Kenya [50]. From the overall satisfaction perspective, we could conclude that some positive effects had been achieved by the rural BPHSs’ delivery system in China however, there is still much to be improved for rural patients’ overall satisfaction rate or satisfaction score. In particular, the domains among rural BPHSs with low resident satisfaction scores should be paid more attention.

This study indicates that PHC worker’s attitude was identified as the domain with the highest satisfaction score among rural residents, and it was also the strongest influencing factor associated with residents’ overall satisfaction with rural BPHSs. A high satisfaction with the attitude of PHC workers would bring a high overall satisfaction of residents with rural BPHSs. The findings are in accordance with results in previous studies in China. Xu et al. [37] and Liu et al. [40] revealed that 98.76% and 92.43% of rural residents were satisfied with PHC workers’ attitude in the provision of BPHSs in Zhejiang and Gansu, respectively, both of which were higher than the satisfaction rates with BPHSs items. These results suggest that besides BPHSs’ items, rural patients also care a lot about PHC worker attitudes, from which they might feel respect, politeness, etc. [21].

Immunization and health management for children was detected as the domain with the highest satisfaction score of rural residents in rural BPHSs’ items, but in spite of this, the score was low. Meanwhile, our study further found that a high satisfaction with immunization and health management for children could significantly contribute to a high residents’ overall satisfaction with rural BPHSs. Similar findings have been reported in prior studies. Xu et al. [45], Zhang et al. [46], and Shang et al. [42] found rural residents were most satisfied with immunization and health management for children in Anhui, Shandong, and Zhejiang, respectively. This might be related to the better implementation effect of immunization and health management for children than other BPHSs items. At the end of 2016, more than 95% of children received immunization services, and approximately 90% of children aged three years or below were provided with health management in China [51].

The study found that health management for rural residents with chronic diseases was the least satisfied domain among the BPHSs items, and had a significant positive association with rural residents’ overall satisfaction with BPHSs. The result was consistent with the existing evidence [42,45,46]. For example, Xu et al. found that only 21.63% of rural residents were satisfied with the health management services for chronic diseases [45]. These results imply that the health management for rural residents does not work well in China. At the end of 2016, only 70.31% of residents with hypertension and 65.57% of residents with diabetes were provided with standardized health management [51].

Meanwhile, in terms of other rural BPHSs, i.e., health education, maternal health management, elderly health management, and reporting and handling of infectious diseases, residents reported low satisfaction scores that ranged from 3.40 to 3.50 out of a maximum of 5. Meanwhile, they were identified to have significant positive association with rural residents’ overall satisfaction with BPHSs. The rural resident satisfaction scores on these BPHSs could still be improved.

In addition, the age of rural residents was identified to be significantly associated with their overall satisfaction with the BPHSs. Older rural residents presented a higher satisfaction with BPHSs. Compared with those who were ≤35 years, residents who were ≥46 years were 1.33 times more likely to be satisfied with rural BPHSs. The potential explanations include that the attitudes of PHC workers might be better toward the relatively older residents and these residents might have a lower expectation of BPHSs [21]. However, in terms of other characteristic indicators, i.e., gender, education, occupation, monthly income, and health status, there was no significant association between them and overall satisfaction with rural BPHSs.

There are several implications. First, in order to further improve the utilization of rural BPHSs, rural residents’ health consciousness and their cooperation should be improved. Higher quality and more attractive health education activities should be implemented. Secondly, based on the findings in our study, the shortage of PHC workers in rural areas should be addressed. Rural medical institutions could recruit more medical graduates who major in public health and graduate from junior medical colleges or below. On the one hand, they can provide more professional services in the field of public health and on the other hand, these graduates are more likely to work in rural areas compared with those in medical universities [52]. Thirdly, there is still a large area for improvement in regards to rural resident satisfaction with BPHSs. For the sake of the improvement of residents’ satisfaction, it should further strengthen the coverage and implementation quality of these rural BPHSs, especially health management for rural residents with chronic diseases like hypertension and diabetes. Additionally, we advise that resident satisfaction with BPHSs should be included in the performance assessment system for PHC workers.

Finally, some limitations that exist in the study should be acknowledged. Firstly, we did include all 12 BPHSs’ categories in the study, which means the findings in this study could not fully reflect rural resident experiences with BPHSs. Secondly, some BPHSs in China are provided only for specific groups of the population like the elderly, women, children, and residents with chronic diseases and participants who do not have direct experience on these services were asked to report their feeling based on the experience of their families, friends, or neighbors, which might bias results of this study to some extent. Thirdly, although the sample size of this study is large, as it covers a very large group of the rural population in Western China, some results in this study might not parallel the experience with BPHSs of the whole rural population in western areas, and they might not apply well to the rural population in other regions of China. Fourth, although the survey was totally anonymous, because the invited residents knew that the survey was under permission and support by medical institutions, they might be inclined to report high satisfaction ratings with rural RTMSs, which might bring bias to the results. Finally, as this is a cross-sectional design, all the association between the influencing factors identified in the study and residents’ overall satisfaction with rural BPHSs could not be concluded as the causal relationship.

## 5. Conclusions

The findings of this study could provide evidence for the progress of China’s new health-care reform and the growing body of studies on public health from the view of rural residents utilizing the services. The study implied that both the rural residents’ overall satisfaction rate and satisfaction score could still be improved to a large extent. Health-related policy makers should press ahead with the implementation of BPHSs, especially in rural areas. The utilization of rural BPHSs by residents should be improved through improving rural residents’ health consciousness and addressing the shortage of rural PHC workers. Rural residents’ satisfaction with BPHSs should be ameliorated by further improving the coverage and quality of each of the BPHSs’ categories, especially the health management of rural residents with chronic diseases. We also suggest the inclusion of resident satisfaction with the performance assessment system in provision of BPHSs for PHC workers. In general, a resident-centered BPHSs delivery system in rural areas of China should be strengthened.

## Figures and Tables

**Table 1:** Participants’ characteristics.

Characteristics	N	%
**Gender (*n* = 8949)**		
Female	5061	56.55
Male	3888	43.45
**Age (*n* = 8632)**		
≤35 years	3658	42.38
36–45 years	1795	20.79
≥46 years	3179	36.83
**Education (*n* = 8980)**		
Illiteracy	1336	14.88
Primary school	2036	22.67
Junior high school	2770	30.85
Senior high school and above	2838	31.6
**Occupation (*n* = 8984)**		
Farmer	4223	47.01
Nonfarmer	3603	40.1
Other	1158	12.89
**Monthly income (*n* = 8956)**		
No regular income	2796	31.22
≤2000 Yuan	3754	41.92
≥2001 Yuan	2406	26.86
**Health status (*n* = 9019)**		
Relatively bad	2371	26.29
Good	6648	73.71

**Table 2:** Resident satisfaction with rural basic public health services.

Indicators	Likert 5-point Scale of Resident Satisfaction, N (%)					Satisfaction Score	Spearman Correlation ^1^	
	Strongly Dissatisfied	Dissatisfied	Neither Dissatisfied nor Satisfied	Satisfied	Strongly Satisfied	Mean ± S.D. ^2^	Coeffi. ^3^	*p*-Value
Health education	198 (2.20)	1314 (14.63)	3288 (36.61)	3103 (34.55)	1078 (12.00)	3.40 ± 0.951	0.537	<0.001
Immunization and health man. ^4^ for children	135 (1.50)	997 (11.10)	2848 (31.69)	3698 (41.15)	1308 (14.56)	3.56 ± 0.922	0.521	<0.001
Maternal health man. ^4^	113 (1.26)	1223 (13.61)	2990 (33.29)	3398 (37.83)	1259 (14.02)	3.50 ± 0.937	0.579	<0.001
Elderly health man. ^4^	135 (1.50)	1557 (17.30)	3026 (33.63)	3109 (34.55)	1172 (13.02)	3.40 ± 0.968	0.577	<0.001
Health man. ^4^ for residents with chronic diseases	153 (1.70)	1729 (19.23)	2890 (32.15)	2979 (33.14)	1239 (13.78)	3.38 ± 0.999	0.591	<0.001
Reporting and handling of infectious diseases	132 (1.47)	1540 (17.13)	2955 (32.87)	3129 (34.81)	1233 (13.72)	3.42 ± 0.974	0.592	<0.001
PHC worker’s attitude	132 (1.47)	764 (8.49)	2817 (31.29)	3759 (41.75)	1532 (17.01)	3.64 ± 0.910	0.710	<0.001
Overall satisfaction	124 (1.37)	813 (9.01)	2950 (32.71)	3666 (40.65)	1466 (16.25)	3.61 ± 0.908	N/A	

^1^ spearman correlation with overall satisfaction. ^2^ S.D. refers to standard deviation. ^3^ coeffi. refers to coefficient. ^4^ health man. refers to health management.

**Table 3:** Overall satisfaction difference among residents with different characteristics.

Characteristics	Mean ± S.D. ^1^	One-Way ANOVA
		*F*-Value (*p*-Value)
**Gender**		1.092 (0.296)
Female	3.62 ± 0.888	
Male	3.60 ± 0.932	
**Age**		15.717 (<0.001)
≤35 years	3.55 ± 0.931	
36–45 years	3.64 ± 0.930	
≥46 years	3.67 ± 0.864	
**Education**		2.860 (0.035)
Illiteracy	3.64 ± 0.923	
Primary school	3.65 ± 0.881	
Junior high school	3.59 ± 0.898	
≥ Senior high school	3.59 ± 0.930	
**Occupation**		23.263 (<0.001)
Farmer	3.67 ± 0.874	
Nonfarmer	3.60 ± 0.943	
Other	3.47 ± 0.902	
**Monthly income**		9.711 (<0.001)
No regular income	3.55 ± 0.905	
≤2000 Yuan	3.64 ± 0.909	
≥2001 Yuan	3.64 ± 0.902	
**Health status**		43.336 (<0.001)
Relatively bad	3.72 ± 0.925	
Good	3.58 ± 0.899	

^1^ S.D. refers to standard deviation.

**Table 4:** Multiple linear regression and binary logistic regression analyses on influencing factors of rural residents’ overall satisfaction with basic public health services.

Variables	Multiple Linear Regression				Binary Logistic Regression			
	B (95% CI ^1^)	S.E. ^2^	t-Value	*p*-Value	B	S.E. ^2^	OR ^3^ (95% CI ^1^)	*p*-Value
Satisfaction with health education	0.074 (0.056, 0.092)	0.009	8.160	<0.001	0.323	0.045	1.38 (1.26, 1.51)	<0.001
Satisfaction with immunization and health man. ^4^ for children	0.043 (0.024, 0.061)	0.009	4.498	<0.001	0.170	0.046	1.19 (1.08, 1.30)	<0.001
Satisfaction with maternal health man. ^4^	0.074 (0.054, 0.094)	0.010	7.346	<0.001	0.283	0.048	1.33 (1.21, 1.46)	<0.001
Satisfaction with elderly health man. ^4^	0.073 (0.054, 0.092)	0.010	7.438	<0.001	0.288	0.046	1.33 (1.22, 1.46)	<0.001
Satisfaction with health man. ^4^ for residents with chronic diseases	0.099 (0.080, 0.117)	0.009	10.412	<0.001	0.311	0.044	1.37 (1.25, 1.49)	<0.001
Satisfaction with reporting and handling of infectious diseases	0.128 (0.109, 0.147)	0.010	13.122	<0.001	0.397	0.046	1.49 (1.36, 1.63)	<0.001
Satisfaction with PHC worker’s attitude	0.425 (0.407, 0.443)	0.009	46.171	<0.001	1.527	0.050	4.61 (4.17, 5.08)	<0.001
Age (36–45 years)	0.007 (−0.027, 0.040)	0.017	0.386	0.700	0.074	0.085	1.08 (0.91, 1.27)	0.382
Age (≥46 years)	0.037 (0.005, 0.069)	0.016	2.281	0.023	0.281	0.079	1.33 (1.14, 1.55)	<0.001
Education (Primary school)	0.012 (−0.031, 0.054)	0.022	0.542	0.588	0.119	0.106	1.13 (0.91, 1.39)	0.265
Education (Junior high school)	−0.008 (−0.051, 0.035)	0.022	−0.365	0.715	−0.091	0.107	0.91 (0.74, 1.13)	0.395
Education (≥ Senior high school)	0.002 (−0.046, 0.050)	0.024	0.078	0.938	0.033	0.120	1.03 (0.82, 1.31)	0.782
Occupation (Nonfarmer)	−0.017 (−0.051, 0.018)	0.018	−0.954	0.340	−0.103	0.086	0.90 (0.76, 1.07)	0.227
Occupation (Other)	−0.037 (−0.078, 0.004)	0.021	−1.766	0.077	−0.021	0.102	0.98 (0.80, 1.20)	0.839
Income (≤2000 Yuan)	0.010 (−0.021, 0.040)	0.016	0.640	0.522	0.116	0.076	1.12 (0.97, 1.30)	0.129
Income (≥2001 Yuan)	0.040 (0.003, 0.078)	0.019	2.112	0.035	0.181	0.094	1.20 (0.99, 1.44)	0.055
Health status (Relatively bad)	0.007 (−0.021, 0.036)	0.015	0.509	0.611	0.037	0.073	1.04 (0.90, 1.20)	0.614

^1^ 95% CI refers to 95% confidence interval. ^2^ S.E. refers to standard error. ^3^ OR refers to odds ratio. ^4^ health man. refers to health management.
